# Supplementary material for: Reformulation of an extant ATPase active site to mimic ancestral GTPase activity reveals a nucleotide base requirement for function
Source: eLife. 2021 Mar 11;10:e65845. doi: 10.7554/eLife.65845 (PMC7952092; doi:10.7554/eLife.65845)
Supplement: Supplementary file 1. [file elife-65845-supp1.docx]

**Supplementary File 1.** *Bacillus subtilis* strains used in this study.

| Name | Genotype | | Source | |  |
| --- | --- | --- | --- | --- | --- |
| PY79 | Prototrophic derivative of *B. subtilis* 168 | | (1) | |  |
| KP73 | ∆*spoIVA::neo* | | (2) | |  |
| KR394 | ∆*spoIVA::neo thrC::spoIVA spec* | | (3) | |  |
| NG7 | ∆*spoIVA::neo thrC::spoIVA^S189K^ spec* | | This study | |  |
| NG13 | ∆*spoIVA::neo thrC::spoIVA^R191D^ spec* | | This study | |  |
| NG8 | ∆*spoIVA::neo thrC::spoIVA^S189K, R191D^ spec* | | This study | |  |
| TU209 | ∆*spoIVA::neo thrC::spoIVA^S189K, R191D, S216A, E218A^ spec* | | This study | |  |
| TU210 | ∆*spoIVA::neo thrC::spoIVA^S189K, S216A, E218A^ spec* | | This study | |  |
| TU211 | ∆*spoIVA::neo thrC::spoIVA^S216A, E218A^ spec* | | This study | |  |
| TU212 | ∆*spoIVA::neo thrC::spoIVA^S216A^ spec* | | This study | |  |
| TU213 | ∆*spoIVA::neo thrC::spoIVA^E218A^ spec* | | This study | |  |
| TU223 | ∆*spoIVA::neo thrC::spoIVA^R191D, E218A^ spec* | | This study | |  |
| SL55 | ∆*spoIVA::neo thrC::GFP-spoIVA spec* ∆*amyE::spoIVA cat* | | This study | |  |
| JH19 | ∆*spoIVA::neo thrC::GFP-spoIVA^S189K^ spec* ∆*amyE::spoIVA^S189K^ cat* | | This study | |  |
| JH20 | ∆*spoIVA::neo thrC::GFP-spoIVA^R191D^ spec* ∆*amyE::spoIVA^R191D^ cat* | | This study | |  |
| JH1 | ∆*spoIVA::neo thrC::GFP-spoIVA^S189K, R191D^ spec* ∆*amyE::spoIVA^S189K, R191D^ cat* | | This study | |  |
| TU200 | ∆*spoIVA::neo thrC::GFP-spoIVA^S189K, R191D, S216A, E128A^ spec* ∆*amyE::^spoIVAS189K, R191D, S216A, E218A^ cat* | | This study | |  |
| TU201 | ∆*spoIVA::neo thrC::GFP-spoIVA^S216A, E218A^ spec* ∆*amyE::spoIVA^S216A, E218A^ cat* | | This study | |  |
| TU202 | ∆*spoIVA::neo thrC::GFP-spoIVA^S216A^ spec* ∆*amyE::spoIVA^S216A^ cat* | | This study | |  |
| TU203 | ∆*spoIVA::neo thrC::GFP-spoIVA^E218A^ spec* ∆*amyE::spoIVA^E218A^ cat* | | This study | |  |
| TU227 | ∆*spoIVA::neo thrC::GFP-spoIVA^R191D, E218A^ spec* ∆*amyE::spoIVA^R191D, E218A^ cat* | | This study | |  |
|  |  |  | |  | |

1. Youngman P, Perkins JB, Losick R. Construction of a cloning site near one end of Tn917 into which foreign DNA may be inserted without affecting transposition in *Bacillus subtilis* or expression of the transposon-borne erm gene. *Plasmid.* 1984;12: 1-9.
2. Price KD, Losick R. A four-dimensional view of assembly of a morphogenetic protein during sporulation in *Bacillus subtilis*. *J Bacteriol*. 1999;181: 781-790.
3. Ramamurthi KS, Losick R. ATP-driven self-assembly of a morphogenetic protein in *Bacillus subtilis*. *Mol Cell*. 2008:31: 406-414.
