# Supplementary material for: Reformulation of an extant ATPase active site to mimic ancestral GTPase activity reveals a nucleotide base requirement for function
Source: eLife. 2021 Mar 11;10:e65845. doi: 10.7554/eLife.65845 (PMC7952092; doi:10.7554/eLife.65845)
Supplement: Supplementary file 2. — For subcellular localization data, ‘+' indicates forespore localization pattern qualitatively similar to wild-type GFP-SpoIVA; ‘-' indicates mis-localization. Errors are S.D. [file elife-65845-supp2.docx]

**Supplementary File 2. Summary of results from in vitro and in vivo assays performed with various SpoIVA variants. For subcellular localization data, “+” indicates forespore localization pattern qualitatively similar to wild type GFP-SpoIVA; “-” indicates mis-localization. Errors are S.D.**

|  |  |  |  |  | **SpoIVA** | |  | |  |  | |  | | |  |  |
| --- | --- | --- | --- | --- | --- | --- | --- | --- | --- | --- | --- | --- | --- | --- | --- | --- |
| ***In vitro assays*** | **NSxR SxE** | **NSxD SxE** | **NKxR SxE** | **NKxD SxE** | **NKxD AxA** | | **NSxR AxA** | | **NSxR AxE** | **NSxR SxA** | | **NSxD SxA** | | |  |  |
| *K*_m_ (mM) +ATP | 0.52±0.12 | 0.53±0.06 | 1.08±0.22 | 1.15±0.16 | 1.48±0.39 | 2.53±1.01 | | 1.91±0.91 | | 3.78±1.02 | | 3.92±1.02 | | |  |  |
| *K*_m_ (mM) +GTP | 1.03±0.31 | 1.79±0.12 | 2.31±0.7 | 2.5±0.69 | 1.87±0.87 | 2.64±0.82 | | 1.63±1.28 | | 3.01±1.87 | | 2.44±0.89 | | |  |  |
| *K*_cat._ (min-^1^) +ATP | 2.14±0.64 | 1.78±0.17 | 13.60±0.42 | 11.6±0.95 | 6.28±2.69 | 6.17±1.89 | | 5.48±1.27 | | 4.89±1.55 | | 1.90±0.69 | | |  |  |
| *K*_cat._ (min^-1^) +GTP | 1.15±0.47 | 1.14±0.28 | 10.90±1.14 | 11.00±0.08 | 4.04±2.71 | 5.76±2.71 | | 2.78±1.36 | | 2.85±1.69 | | 1.30±0.29 | | |  |  |
| Cat. Eff. (min^-1^ mM^-1^)  +ATP | 4.2±0.9 | 3.4±0.7 | 13.28±2.8 | 10.2±1.1 | 4.3±1.4 | 3.1±1.2 | | 3.6±2.0 | | 1.5±0.3 | | 0.46±0.04 | | |  |  |
| Cat. Eff. (min^-1^ mM^-1^)  +GTP | 1.3±0.8 | 0.6±0.1 | 5.1±1.9 | 4.6±1.1 | 2.1±0.8 | 2.2±1.0 | | 2.1±1.5 | | 1.0±0.3 | | 0.63±0.3 | | |  |  |
| Cat. Eff. Ratio  (+ATP/+GTP) | 3.6±1.4 | 5.6±1.9 | 2.4±0.5 | 2.1±0.4 | 2.0±0.6 | 1.4±0.4 | | 2.0±0.8 | | 1.4±0.4 | | 0.8±0.25 | | |  |  |
| Trypsin digest, ATP  (Decay rate, +/- ATP) | 0.22±0.05 | 0.25±0.01 | 1.03±0.12 | 0.97±0.06 | 1.01±0.16 | 0.55±0.04 | | 0.44±0.14 | | 0.53±0.03 | | 0.35±0.19 | | |  |  |
| Trypsin digest, GTP  (Decay rate, +/- GTP) | 0.27±0.09 | 0.34±0.04 | 1.1±0.2 | 0.99±0.12 | 1.1±0.11 | 0.59±0.06 | | 0.47±0.03 | | 0.52±0.07 | | 0.4±0.22 | | |  |  |
| Polymerization  (Rate +/- ATP) | 32.97±16.46 | 26.58±21.01 | 0.69±0.08 | 1.74±1.77 | 0.89±0.38 | 8.73±3.29 | | 17.33±1.30 | | 7.36±1.8 | | 13.71±8.35 | | |  |  |
| Polymerization  (Rate +/- GTP) | 6.24±2.77 | 0.92±0.41 | 0.67±0.03 | 1.1±0.47 | 0.87±0.28 | 1.31±0.67 | | 1.15±0.93 | | 2.13±1.17 | | 1.49±0.88 | | |  |  |
|  |  |  |  |  |  | |  | |  | |  | |  |  |  | |
| ***In vivo assays*** |  |  |  |  |  | |  | |  | |  | |  |  |  |  |
| Sporulation Efficiency relative to WT  (heat resistance) | 0.77±0.33 | 0.86±0.15 | >10^-8^ | >10^-3^ | >10^-6^ | | 0.69±0.33 | | 0.82±0.34 | | 0.69±0.15 | | 0.74±0.22 |  |  | |
| Sporulation Efficiency relative to WT  (lysozyme resistance) | 0.69±0.09 | 0.77±0.02 | Not determined | >10^-2^ | >10^-4^ | | 0.44±0.05 | | 0.61±0.09 | | 0.83±0.05 | | 0.74±0.01 |  |  | |
| Localization to forespore | + | + | - | - | - | | + | | + | | + | | + |  |  | |
|  |  |  |  |  |  | |  | |  | |  | |  |  |  | |
