# Supplementary material for: Reformulation of an extant ATPase active site to mimic ancestral GTPase activity reveals a nucleotide base requirement for function
Source: eLife. 2021 Mar 11;10:e65845. doi: 10.7554/eLife.65845 (PMC7952092; doi:10.7554/eLife.65845)
Supplement: Supplementary file 3. — 0 hr time point indicates time of sporulation induction by resuspension; pre-induction is immediately prior to resuspension. Errors are S.D. [file elife-65845-supp3.docx]

**Supplementary File 3. Nucleotide levels (ion counts) at indicated time points after induction of sporulation in *B. subtilis* via resuspension method. 0 h time point indicates time of sporulation induction by resuspension; pre-induction is immediately prior to resuspension. Errors are S.D.**

**Time post-induction of sporulation (h)**

| Nucleotide | Pre-induction | 0 | 1 | 2 | 3.5 | 5 |
| --- | --- | --- | --- | --- | --- | --- |
| ATP | 1.1×10^9^ ± 4.2×10^8^ | 1.2×10^9^ ± 4.5×10^8^ | 2.2×10^9^ ± 2.1×10^7^ | 1.2×10^9^ ± 5.7×10^7^ | 7.8×10^8^ ± 1.2 ×10^8^ | 3.8×10^8^ ± 4.0×10^7^ |
| ADP | 7.6×10^8^ ± 6.3×10^7^ | 1.5×10^9^ ±  2.9×10^8^ | 1.3×10^9^ ±  2.3×10^7^ | 7.9×10^8^ ±  5.1×10^7^ | 5.4×10^8^ ±  6.5×10^8^ | 4.4×10^8^ ±  6.2×10^7^ |
| AMP | 2.9×10^8^ ±  3.5×10^7^ | 5.2×10^8^ ±  1.1×10^8^ | 6.3×10^8^ ±  1.0×10^8^ | 3.4×10^8^ ±  2.2×10^7^ | 2.7×10^8^ ±  2.9×10^7^ | 2.9×10^8^ ±  1.5×10^7^ |
| GTP | 3.1×10^8^ ±  1.6×10^8^ | 3.4×10^7^ ±  1.6×10^7^ | 4.8×10^7^ ±  7.7×10^6^ | 6.7×10^7^ ±  9.5×10^6^ | 9.6×10^7^ ±  1.6×10^7^ | 7.5×10^7^ ±  5.7×10^6^ |
| GDP | 2.8×10^8^ ±  1.5×10^8^ | 6.1×10^7^ ±  1.8×10^7^ | 4.8×10^7^ ±  7.7×10^6^ | 6.5×10^7^ ±  1.0×10^7^ | 7.4×10^7^ ±  1.2×10^7^ | 8.3×10^7^ ±  3.8×10^6^ |
| GMP | 2.3×10^7^ ±  1.0×10^7^ | 3.1×10^7^ ±  1.3×10^7^ | 7.5×10^6^ ±  1.8×10^6^ | 5.9×10^6^ ±  2.3×10^6^ | 2.5×10^7^ ±  7.2×10^6^ | 8.7×10^7^ ±  1.2×10^7^ |
| CTP | 2.6×10^8^ ±  4.0×10^7^ | 2.8×10^8^ ±  1.0×10^8^ | 4.4×10^8^ ±  1.5×10^7^ | 2.3×10^8^ ±  2.1×10^7^ | 1.6×10^8^ ±  1.0×10^7^ | 1.5×10^8^ ±  1.8×10^7^ |
| CDP | 7.9×10^7^ ±  7.8×10^6^ | 2.2×10^8^ ±  6.4×10^7^ | 2.7×10^8^ ±  3.0×10^7^ | 1.4×10^8^ ±  9.2×10^6^ | 1.0×10^8^ ±  1.2×10^7^ | 1.3×10^8^ ±  1.1×10^7^ |
| CMP | 3.1×10^7^ ±  2.2×10^6^ | 1.0×10^8^ ±  7.5×10^6^ | 5.6×10^7^ ±  3.6×10^6^ | 2.9×10^7^ ±  2.9×10^6^ | 4.6×10^7^ ±  5.6×10^6^ | 1.1×10^8^ ±  2.1×10^7^ |
| UTP | 5.1×10^8^ ±  6.0×10^7^ | 2.0×10^8^ ±  4.9×10^7^ | 4.6×10^8^ ±  1.7×10^7^ | 2.8×10^8^ ±  1.8×10^7^ | 2.3×10^8^ ±  1.7×10^7^ | 1.8×10^8^ ±  1.8×10^7^ |
| UDP | 2.8×10^8^ ±  4.5×10^7^ | 2.8×10^8^ ±  6.2×10^7^ | 4.4×10^8^ ±  3.9×10^7^ | 2.6×10^8^ ±  2.0×10^7^ | 2.1×10^8^ ±  1.9×10^7^ | 2.2×10^8^ ±  1.9×10^7^ |
| UMP | 1.2×10^8^ ±  8.4×10^7^ | 2.9×10^8^ ±  1.6×10^7^ | 1.4×10^8^ ±  6.7×10^6^ | 6.8×10^7^ ±  4.1×10^6^ | 8.9×10^7^ ±  1.8×10^7^ | 1.7×10^8^ ±  3.8×10^7^ |
| ppGpp | 1.9×10^6^ ±  3.3×10^6^ | 5.4×10^7^ ±  4.2×10^7^ | 1.8×10^7^ ±  8.4×10^6^ | 1.2×10^5^ ±  6.6×10^4^ | 2.8×10^4^ ±  4.0×10^4^ | 2.5×10^4^ ±  2.3×10^4^ |
| pGpp | 6.3×10^6^ ±  5.1×10^6^ | 1.1×10^8^ ±  5.9×10^7^ | 4.2×10^7^ ±  1.3×10^7^ | 8.8×10^6^ ±  9.1×10^5^ | 5.1×10^6^ ±  9.7×10^5^ | 4.5×10^6^ ±  7.5×10^5^ |
