# Supplementary material for: Reformulation of an extant ATPase active site to mimic ancestral GTPase activity reveals a nucleotide base requirement for function
Source: eLife. 2021 Mar 11;10:e65845. doi: 10.7554/eLife.65845 (PMC7952092; doi:10.7554/eLife.65845)
Supplement: Transparent reporting form [file elife-65845-transrepform.docx]

***eLife’s* transparent reporting form**

We encourage authors to provide detailed information *within their submission* to facilitate the interpretation and replication of experiments. Authors can upload supporting documentation to indicate the use of appropriate reporting guidelines for health-related research (see [EQUATOR Network](http://www.equator-network.org/%20)), life science research (see the [BioSharing Information Resource](https://biosharing.org/)), or the [ARRIVE guidelines](http://www.plosbiology.org/article/info:doi/10.1371/journal.pbio.1000412) for reporting work involving animal research. Where applicable, authors should refer to any relevant reporting standards documents in this form.

If you have any questions, please consult our Journal Policies and/or contact us: [editorial@elifesciences.org](mailto:editorial@elifesciences.org).

**Sample-size estimation**

- You should state whether an appropriate sample size was computed when the study was being designed
- You should state the statistical method of sample size computation and any required assumptions
- If no explicit power analysis was used, you should describe how you decided what sample (replicate) size (number) to use

Please outline where this information can be found within the submission (e.g., sections or figure legends), or explain why this information doesn’t apply to your submission:

All experiments reported were either computational (phylogenetic analyses), in vitro activity assays (NTP hydrolysis, polymerization, trypsin digest) using purified proteins), or in vivo functionality using bacterial cultures, which did not involve computing sample sizes.

**Replicates**

- You should report how often each experiment was performed
- You should include a definition of biological versus technical replication
- The data obtained should be provided and sufficient information should be provided to indicate the number of independent biological and/or technical replicates
- If you encountered any outliers, you should describe how these were handled
- Criteria for exclusion/inclusion of data should be clearly stated
- High-throughput sequence data should be uploaded before submission, with a private link for reviewers provided (these are available from both GEO and ArrayExpress)

Please outline where this information can be found within the submission (e.g., sections or figure legends), or explain why this information doesn’t apply to your submission:

Sporulation efficiency data.

Sporulation efficiency was measured as survival after heat treatment of three independent bacterial cultures, along with standard deviation.

Fig. 3A legend: “(n=3-4); bars indicate mean values; error bars are S.D.; “<10^-8^” indicates that no heat-resistant spores were recovered. Sporulation efficiencies are listed in Table S2.” Data from each replicate is shown as a data point, so that the reader may appreciate the spread of data and how mean values were calculated.

Biochemistry experiments.

Nucleotide hydrolysis assays.

Fig. 2A legend: “Each data point represents mean results of an independent assay performed 3-4 times with one batch of purified protein; bars represent aggregate mean values from all experiments (also stated above each data set); error bars are S.D.” Additionally, individual representative Michaelis-Menten saturation curves are presented in Fig. S1; Table S2 contains computed values; also included is a source data file that contains all enzyme parameters used to calculate the data in Fig. 2A.

Limited trypsin digest assays.

Fig. 4A legend: “Displayed is a representative image (n=3-4)”. Calculated decay values for full length protein (displayed in Fig. 4B), with S.D. are reported in Table S2.

Fig. 4C-F legend: “Data points represent decay rate ratios from independent assays (n=3-4); bars indicate mean values; error bars are S.D.” Gels used to calculate these values are displayed in Fig. S4.

Dynamic light scattering assays.

Fig. 6G legend: “Each data point represents a ratio obtained from independent assays (n=3) in the presence and absence of ATP; bars represent mean values; error bars are S.D.”

In vivo nucleotide concentration determination.

Fig. 6A-D legend: “Data points indicate mean (n = 3 independent cultures); error bars are S.E.M. Ion count values are listed in Table S3.”

**Statistical reporting**

- Statistical analysis methods should be described and justified
- Raw data should be presented in figures whenever informative to do so (typically when N per group is less than 10)
- For each experiment, you should identify the statistical tests used, exact values of N, definitions of center, methods of multiple test correction, and dispersion and precision measures (e.g., mean, median, SD, SEM, confidence intervals; and, for the major substantive results, a measure of effect size (e.g., Pearson's r, Cohen's d)
- Report exact p-values wherever possible alongside the summary statistics and 95% confidence intervals. These should be reported for all key questions and not only when the p-value is less than 0.05.

Please outline where this information can be found within the submission (e.g., sections or figure legends), or explain why this information doesn’t apply to your submission:

Fig. 2A legend: Each data point represents mean results of an independent assay performed 3-4 times with one batch of purified protein; bars represent aggregate mean values from all experiments (also stated above each data set); error bars are S.D.

Fig. 2B legend: Data points represent ratios obtained from an independent parallel assay using ATP and GTP; bars represent mean values (also stated above each data set); error bars are S.D.

Fig. 3A legend: Data points represent sporulation efficiencies from independent cultures (n=3-4); bars indicate mean values; error bars are S.D.

Fig. 4A legend: Displayed is a representative image (n=3-4)

Fig. 4B-F legend: Data points represent decay rate ratios from independent assays (n=3-4); bars indicate mean values; error bars are S.D.

Fig. 5A legend: bars represent mean values; error bars are S.D.

Fig. 5C legend: Depicted is a single representative experiment that was performed three times.

Fig. 6A-D legend: Data points indicate mean (n = 3 independent cultures); error bars are S.E.M.

Fig. 6E legend: Displayed is a representative experiment (n=3-5)

Fig. 6F legend: Each point represents an independent experiment (n=3-5)

Fig. 6G legend: Each data point represents a ratio obtained from independent assays (n=3) in the presence and absence of ATP; bars represent mean values; error bars are S.D.

Fig. S1 legend: Catalytic efficiencies derived from calculated *k*_cat_ and *K*_m_ values represent one data point plotted in Fig. 2A and reported in Table S2. Error bars represent S.D. (n=3).

Fig. S3 legend: Data points represent mean values (n=3); error bars are S.D. The ratio of the calculated polymerization rate for samples with ATP or GTP over no NTP for each curve represents one data point in Fig. 5A.

Fig. S4 legend: Displayed is a representative image (n=3-4).

(For large datasets, or papers with a very large number of statistical tests, you may upload a single table file with tests, Ns, etc., with reference to sections in the manuscript.)

**Group allocation**

- Indicate how samples were allocated into experimental groups (in the case of clinical studies, please specify allocation to treatment method); if randomization was used, please also state if restricted randomization was applied
- Indicate if masking was used during group allocation, data collection and/or data analysis

Please outline where this information can be found within the submission (e.g., sections or figure legends), or explain why this information doesn’t apply to your submission:

Not applicable for bacterial image analyses in which isogenic strains were used.

Not applicable for in vitro biochemistry analyses in which purified proteins were used and experiments were performed at least in triplicate.

**Additional data files (“source data”)**

- We encourage you to upload relevant additional data files, such as numerical data that are represented as a graph in a figure, or as a summary table
- Where provided, these should be in the most useful format, and they can be uploaded as “Source data” files linked to a main figure or table
- Include model definition files including the full list of parameters used
- Include code used for data analysis (e.g., R, MatLab)
- Avoid stating that data files are “available upon request”

Please indicate the figures or tables for which source data files have been provided:

Source data file: intracellular nucleotide levels. Contains ion counts for all measured metabolites.

Source data file: in vitro assays. Contains calculated values for Km, kcat, catalytic efficiency, decay values for limited trypsin digest, and polymerization rates.

Source data file: raw data for enzyme kinetics. Contains data points for each protein variant to reconstruct Michaelis-Menten curves.
